# Stability of Graphene/Intercalated Oxygen/Ru(0001) as Studied by Thermal Desorption of CO and CO_2_ Molecules

**DOI:** 10.3390/molecules28062670

**Published:** 2023-03-15

**Authors:** Xiaofeng Yu, Steinar Raaen

**Affiliations:** Physics Department, Norwegian University of Science and Technology (NTNU), N7491 Trondheim, Norway

**Keywords:** Ru(0001), graphene, ethylene, oxidation, XPS, UPS, LEED, TPD

## Abstract

Formation of graphene on Ru(0001) by exposure to ethylene and subsequent annealing has been studied by low-energy electron diffraction, X-ray photoelectron spectroscopy, and ultraviolet photoelectron spectroscopy. The stability of graphene/intercalated oxygen/Ru(0001) has been investigated by temperature programmed desorption spectroscopy. Desorption of CO and CO_2_ was observed upon heating the samples to temperatures above 700 K. It was found that the graphene layer was partly intact after the desorption run and that the intercalated oxygen was removed. It was concluded that the oxygen-intercalated graphene layer was stable up to temperatures of about 700 K.

## 1. Introduction

Ever since the publication of a method to prepare free-standing graphene [1], a large amount of scientific activity has been devoted to the topic. The unique electronic properties and high stability of graphene layers due to strong sp${}^{2}$ hybridized bonds have triggered a large interest in the scientific community. The electronic structure of the two-dimensional material shows a linear dispersion near the Dirac point which gives rise to exotic electronic transport properties and high charge carrier concentrations which is relevant for electronic devices [2,3]. In addition to the interesting electronic properties, graphene coatings have been used as protective layers on several metal surfaces inhibiting surface oxidation in ambient atmospheres [4,5,6,7,8,9,10,11,12,13]. Graphene on Ru(0001) was found to provide good protection towards exposure to atmospheric conditions in the case of a complete graphene layer at ambient temperatures [6], however, the graphene on the Ru system could be readily oxidized at temperatures above 600 K [14]. A single layer of graphene can provide efficient protection from oxidation at ambient conditions due to a high energy barrier to oxygen mobility from the surface to the reactive metal layer below [8].

One particular aspect in several studies has been to control the interaction between the graphene layer and the substrate. This may be achieved by using different substrates but also by the intercalation of atoms between the substrate and graphene layer. The focus of the present study is to investigate the temperature stability of the graphene layer in a system where oxygen atoms have been intercalated between the substrate and the graphene layer. Formation of graphene on metallic substrates may be obtained by heating so that carbon impurities in the bulk segregate to the surface [15,16,17] or by exposure to carbon-containing gases at elevated temperatures [18,19,20,21]. Another method of forming graphene is by catalytic graphitisation which uses a carbon containing substrate on which a transition metal is deposited [22]. The graphene layer can be identified by, for example, Scanning Tunneling Microscopy (STM) [3,14,23,24,25,26,27], Low-Energy Electron Diffraction (LEED) [3,23,25,26,27,28,29], or X-ray diffraction (XRD) [25,30]. The present system under investigation is graphene on Ru(0001), which has been extensively studied by a range of experimental techniques, including X-ray photoelectron spectroscopy (XPS) [23,28,31,32], angle resolved photoemission (ARUPS) [31], STM [23,24,29], LEED [23,25], XRD [25,30], He ion-scattering [33], high-resolution electron energy loss spectroscopy (HREELS) [14,34], X-ray photoemission electron microscopy (XPEEM) [32], low-energy electron microsopy (LEEM) [32], Raman spectrocopy [26,35,36], and near-edge X-ray absorption fine structure (NEXAFS) measurements [37]. Theoretical modelling including density functional theory and atomistic modelling [28,38,39,40] has also been performed, which has confirmed the corrugated nature of the graphene layer [23,35]. The strength of chemical bonds between graphene and metal states increases along the series Pt(111), Ir(111), Rh(111), Ru(0001) in which the graphene morphology changes gradually from almost flat to strongly corrugated [37]. The LEED pattern shows a moiré superstructure due to the lattice mismatch between the honeycomb graphene layer and the hexagonal Ru(0001) substrate [23]. The lattice parameter of the graphene lattice is about 92% of the Ru(0001) surface lattice parameter. Two structures (11×11)Ru/(12×12)Graphene [23] and (23×23)Ru/(25×25)Graphene [25] have been proposed. The interaction between the graphene layer and substrate may be changed by intercalation of, for example, oxygen [4,14,29,31] and metal atoms [26,27,32] at the graphene–substrate interface. One aim of studying graphene–metal interfaces is not only to obtain information about the interface, but also to modify the properties of graphene as well as the interface. This may be achieved by adsorption and intercalation of different materials at the interface.

It has been demonstrated that a strongly interacting graphene layer on Ru(0001) may be decoupled from the substrate by intercalation of oxygen atoms. This decoupling results in strong p-doping of the graphene layer [29]. The band structure of the intercalated system has been investigated by angle-resolved photoemission, and it was concluded that the doped system was tunable [29,31]. Presently, the stability of the oxidized graphene/Ru(0001) interface is investigated by thermal desorption measurements of carbon dioxide and carbon monoxide at temperatures up to 1000 K as the graphene layer disintegrates.

## 2. Results and Discussion

### 2.1. Growth of the Graphene/Oxygen/Ruthenium Layer

**Figure 1:**
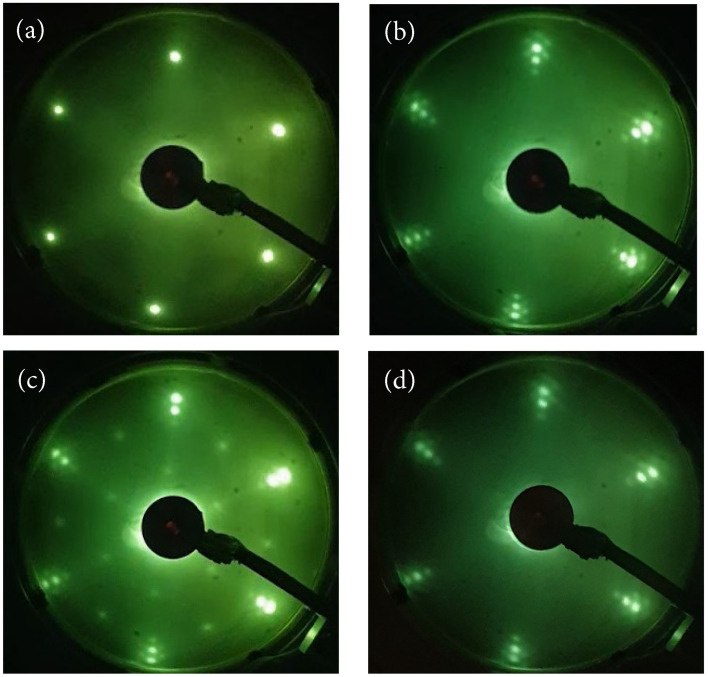
LEED images from: (**a**) Ru(0001); (**b**) Graphene/Ru(0001); (**c**) oxidized graphene/Ru(0001); and (**d**) oxidized sample annealed to 1000 K. An electron beam energy of 64 eV was used.

In the present work, graphene layers were formed by exposure of C_2_H_4_ on Ru(0001) at ambient temperature followed by annealing to 1250 K. Similar results could be obtained by ethylene exposure at temperatures in the range from 1100 to 1250 K. The exposure and annealing cycle was repeated several times to obtain a saturated graphene layer. LEED images are shown in Figure 1: (a) the clean Ru(0001) substrate; (b) a graphene layer on Ru(0001); (c) oxidized graphene layer on Ru(0001); and (d) the oxidized graphene on Ru sample annealed to 1000 K. The electron beam energy was kept at 64 eV for these images. The graphene layer in Figure 1b was produced by exposure to 10 L C_2_H_4_ (1 L(Langmuir) = $1.33\cdot 10^{-4}$ Pa·s) followed by annealing to 1250 K. This exposure and annealing cycle was repeated five times. The LEED image in Figure 1b consists of two hexagons where the larger hexagon corresponds to the graphene layer and the slightly smaller hexagon corresponds to the Ru(0001) substrate. The moiré pattern around each major lattice point is caused by a lattice mismatch, since the graphene lattice parameter is about 92% of the Ru(0001) lattice parameter. By superposing the honeycomb pattern of graphene on the hexagonal Ru(0001) pattern, a large hexagonal pattern appears due to the lattice mismatch [3]. The diffraction pattern in reciprocal space thus produces a small hexagonal pattern around each major LEED spot.

To oxidize the system, the graphene/Ru(0001) sample was exposed to 200 L oxygen gas at a 675 K sample temperature. For lower sample temperatures, the graphene layer acts as a protection against oxidation of the Ru substrate. The corresponding LEED image is shown in Figure 1c, and shows a 2×1 superstructure on the Ru(0001) surface, as previously shown [29,35]. It was concluded that a nearly free-standing character of graphene on Ru(0001) was obtained by oxygen intercalation, and that oxygen formed a saturation phase of 0.5 ML coverage on Ru(0001), which is in agreement with the oxide coverage obtained presently by comparing XPS intensities of the O1s and Ru3d core levels, assuming that the oxide grows at the Ru interface. Subsequent to annealing to 1000 K of the oxidized sample the LEED image in Figure 1d was recorded. The graphene layer seems to be partly intact and the 2×1 pattern has disappeared. However, from the TPD data as well as XPS data after annealing (as presented below) it is clear that the graphene layer is incomplete after annealing to 1000 K. From the LEED images it seems that the sample surface is partly covered by patches of graphene. These patches will be referred to as incomplete graphene layers.

**Figure 2:**
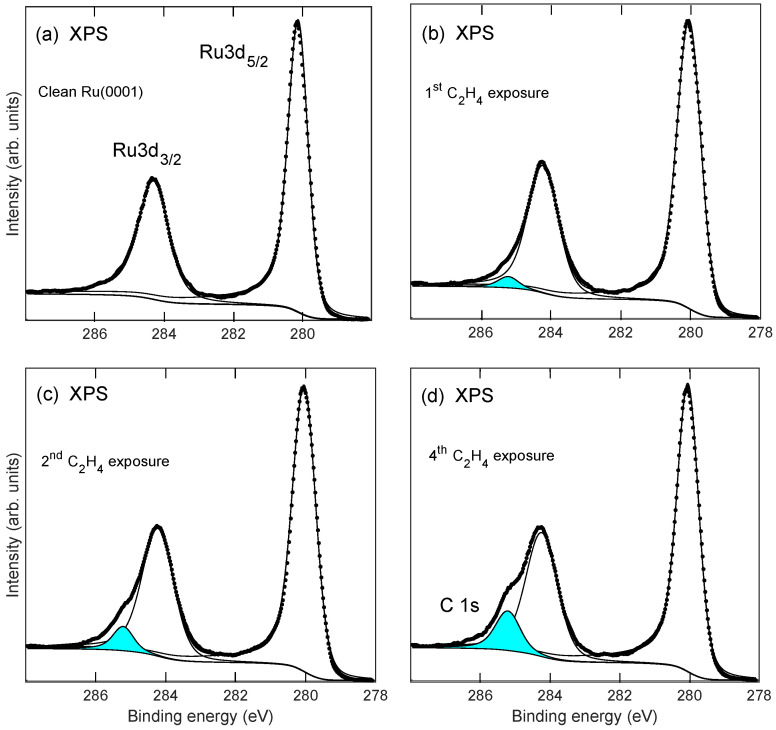
Ru3d${}_{5/2}$, Ru3d${}_{3/2}$, and C1s XPS after multiple ethylene exposure cycles to Ru(0001): Panels (**a**–**d**) show spectra recorded for clean Ru(0001) and after first, second, and fourth exposure cycle, respectively (see text). Solid lines show deconvoluted spectra. The filled peak near-binding energy 285.2 eV in each panel is identified as C 1s emission. The electron emission angle with respect to the surface normal was set at 70° to enhance the surface sensitivity.

**Figure 3:**
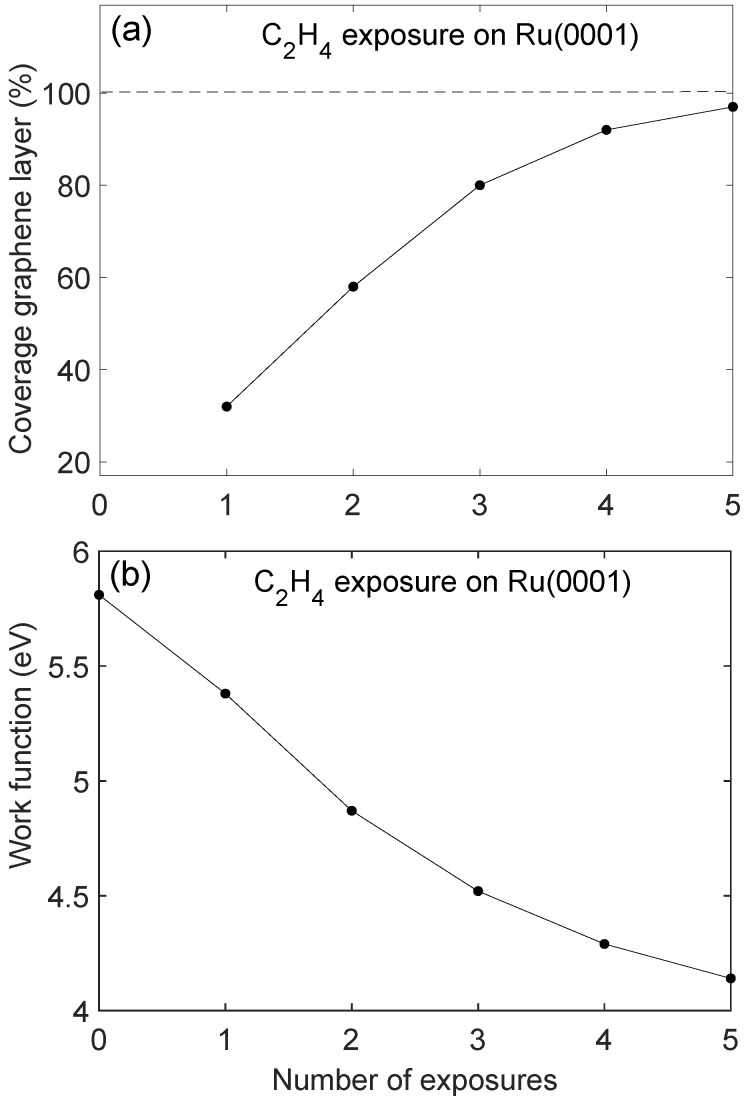
(**a**): Estimated graphene coverage (in % of saturation coverage which is assumed to be one monolayer), and (**b**): Sample work function from UPS data, for increasing number of exposure and annealing cycles of C_2_H_4_ on Ru(0001).

Figure 2 shows XPS data from Ru3d${}_{5/2}$, Ru3d${}_{3/2}$, and C1s core levels for clean Ru(0001) and after multiple ethylene exposure and annealing cycles. The spectra were deconvoluted using the Doniach–Sunjic function [41] which was convoluted by a Gaussian line shape. A Shirley-type background was used which is proportional with the integrated intensity of the core level peak as measured from the low binding energy side of the peak. By deconvoluting the XPS spectra it was possible to extract the C1s emission peak at binding energy near 285.2 eV. It has previously been shown by high-resolution spectra using soft X-ray synchrotron radiation that the C1s emission may be deconvoluted into two components [28,31]. In the present experiments, the resolution and sensitivity were insufficient to resolve these components, and thus, one average component was used for the deconvolution. The peak position of the C1s component thus represents an average over two unresolved components. The deconvoluted spectra were used to estimate the relative peak intensities using core level sensitivity data relevant for the Scienta spectrometer [42]. The XPS data was recorded at an electron exit angle of about 70° with respect to the surface normal to enhance surface sensitivity by a factor of cos(70°). Estimated effective graphene layer coverage as a function of number of ethylene exposure cycles is shown in Figure 3a. After four exposure cycles, the graphene layer thickness seems to be near saturation. The saturated graphene coverage is taken to be one monolayer [28,31]. It was found that the thickness of the graphene layer was over-estimated when determined from XPS intensities. This may partly be due to inaccuracy in determine core level intensities due to the overlap of the C1s and Ru3d levels, and partly due to the possible presence of electron diffraction effects when recording photoelectron intensities at the 70° angle with respect to the surface normal. In Figure 3b the measured work function is shown after each exposure cycle. The measured work function for Ru(0001) is near 5.8 eV and is lowered to near 4.2 eV after five exposure and annealing cycles. The work function value was found to saturate at about 4.0 eV after nine ethylene exposure and annealing cycles. This work function value is lower than that of graphite of about 4.7 eV [43]. As previously reported [35], the lowering of the work function as compared with the value for Ru(0001) as well as graphite indicates strong substrate bonding and significant charge transfer from the metal to the graphene layer.

**Figure 4:**
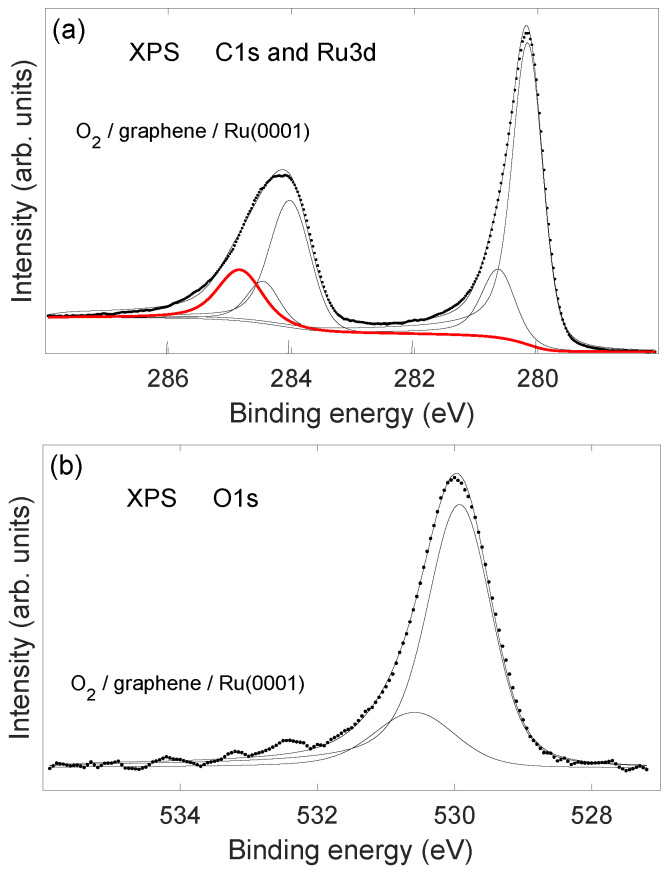
XPS from a saturated graphene layer on Ru(0001) that was exposed to 200 L of oxygen gas at temperature 675 K. (**a**): C1s and Ru3d core levels. The leftmost peak (thick red line) is the C1s emission. (**b**): O1s core level. Solid lines show deconvoluted spectra. The electron exit angle was about 70° with respect to the surface normal.

XPS spectra (recorded using an electron exit angle near 70°) after exposure to 200 L oxygen at temperature 675 K are shown in Figure 4. Panel (a) shows Ru3d and C1s core levels, and (b) shows the O1s core level. The Ru3d spectrum shows two components; the smaller feature to higher binding energy is interpreted to correspond to the Ru-O interface, whereas the major component corresponds to Ru metal. The leftmost peak at binding energy 284.8 eV is the C1s emission from graphene. The change in C1s binding energy from graphene/Ru (285.2 eV) to oxygen/graphene/Ru (284.8 eV) is assumed to reflect weakening of the graphene–substrate interaction as oxygen is intercalated at the interface [29]. Ulstrup et al. [31] published a comprehensive high resolution photoemission study of the intercalation of oxygen in the graphene/Ru(0001) system which show that a dominant C1s peak at binding energy near 283.6 eV appears for the complete oxygen intercalated system for oxygen exposures above $10^{6}$ L. Presently, the oxygen exposure was too small to observe the 283.6 eV component. Deconvolution of the oxygen 1s XPS (panel (b) of Figure 4) shows a smaller peak at the high binding energy side of the main peak. The two components of the O1s core level peak are assumed to represent different oxidation states of Ru at the C-O-Ru interface [44]. The observed binding energy of the O1s XPS in the region 530–531 eV indicates that graphene oxide is not formed during exposure to oxygen of the graphene/Ru system [45].

### 2.2. Stability of Layer

**Figure 5:**
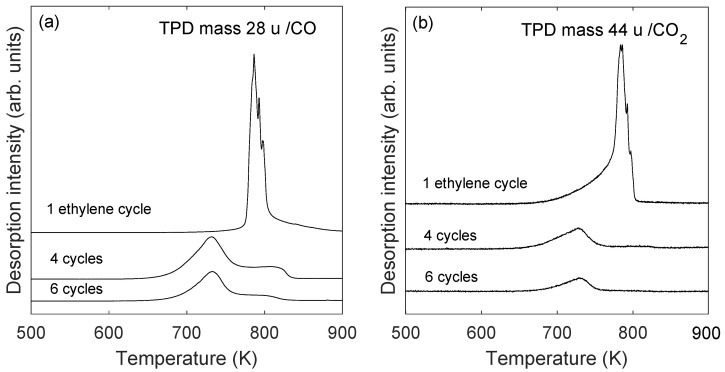
(**a**): TPD of carbon monoxide (mass 28 u), and (**b**) TPD of carbon dioxide (mass 44 u) after exposure to 200 L oxygen at 675 K of graphene/Ru(0001) samples. The ethylene exposure was as indicated on each spectrum (see text).

**Figure 6:**
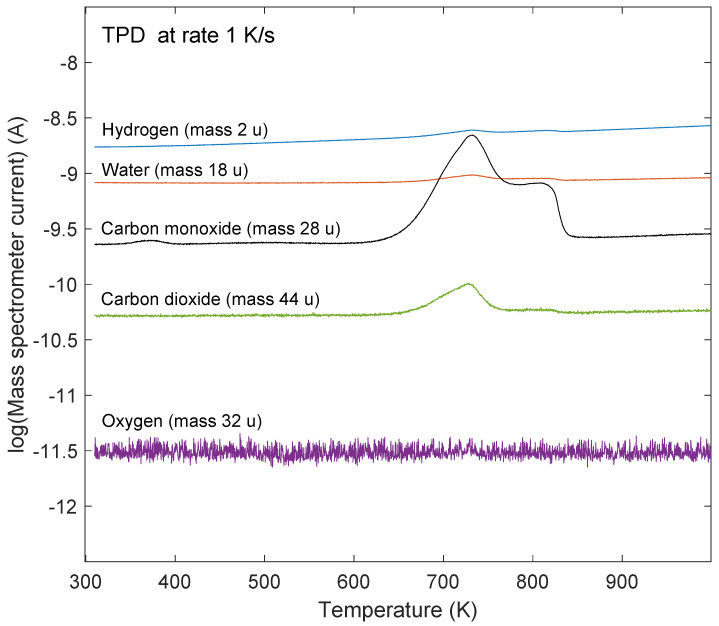
TPD spectra for mass 2, 18, 28, 32, and 44 u after exposure to 200 L oxygen at 675 K of a saturated graphene/Ru(0001) sample.

The stability of the oxidized graphene/Ru(0001) sample was investigated by TPD spectroscopy. The temperature was increased from 300 to 1000 K at a rate of 1 K/s, and desorption of H${}_{2}$ (mass 2 u (1u = 1/12 m${(}^{12}C$)), H_2_O (mass 18 u), CO (mass 28 u), O_2_ (mass 32 u), and CO_2_ (mass 44 u) was monitored. The dominant desorption feature in the recorded spectra is related to CO which is displayed in panel (a) of Figure 5 for 1, 4 and 6 ethylene exposure cycles, as indicated in the figure. In addition to desorption of CO, distinct desorption of CO_2_ was observed. This is shown in panel (b) of Figure 5. The amount of CO_2_ desorption was estimated to be about 5–10% of the amount of CO desorption by taking into account the mass spectrometer sensitivity, which was determined by monitoring the mass spectrometer signal at constant pressure at the ion gauge. These observations indicate that oxygen at the Ru interface reacts with carbon from the graphene layer to desorb as CO and to a lesser extent as CO_2_. It should be noted that no desorption of O_2_ was observed upon heating of the samples to 1000 K, see Figure 6. This is contrary to the conclusion in the work of Li and Yarmoff [33] in which oxygen intercalation between graphene and Ru(0001) was investigated by helium ion scattering. In that work it was claimed that oxygen desorbs in the form of O_2_ which is contradicted by the present work. Figure 6 shows TPD spectra for 2, 18, 28, 44, and 32 amu from a saturated graphene/Ru(0001) sample that was exposed to oxygen. The ordinate axis is plotted on a logarithmic scale.

**Figure 7:**
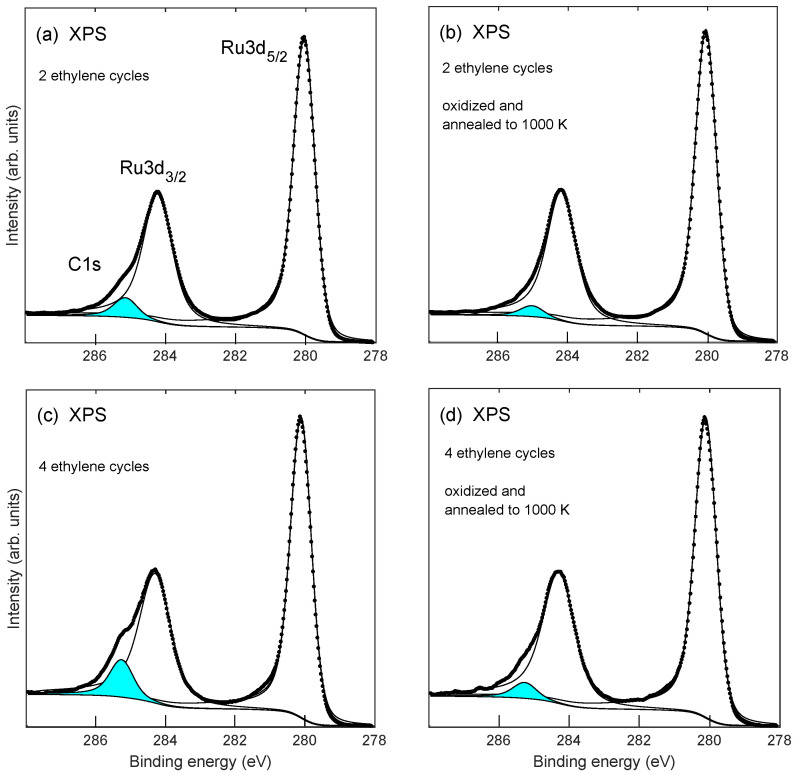
Ru3d and C1s XPS after 2 and 4 ethylene exposure cycles to Ru(0001), panels (**a**,**c**). Panels (**b**,**d**) show spectra that were recorded after annealing to 1000 K of the oxidized systems. Solid lines show deconvoluted spectra. The filled peak near-binding energy 285.2 eV in each panel is identified as C1s emission. The electron emission angle with respect to the surface normal was set at 70° to enhance the surface sensitivity.

**Figure 8:**
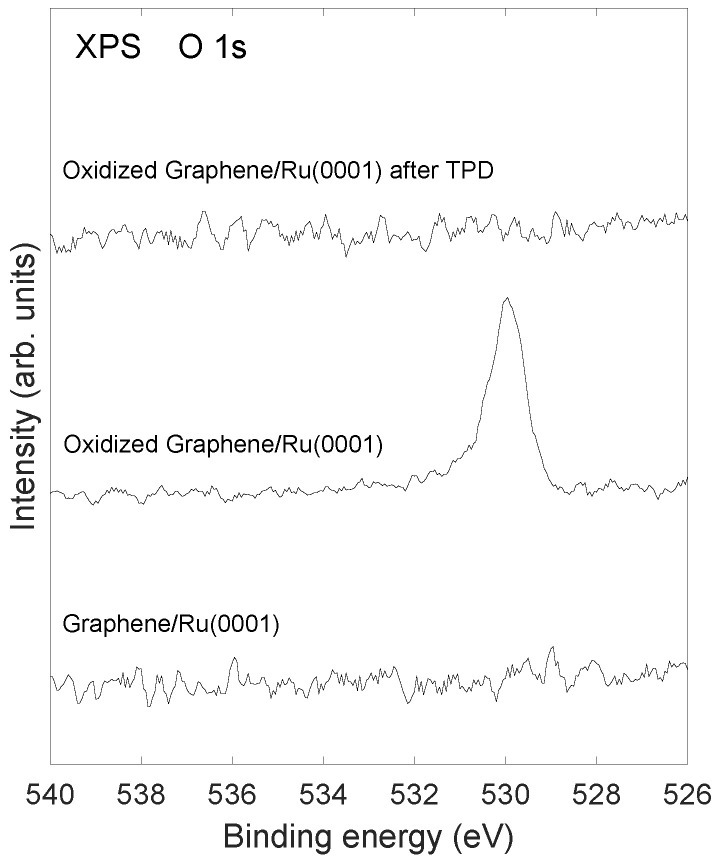
XPS from the O1s core level for: (bottom spectrum) Graphene/Ru(0001); (middle spectrum) oxygen exposed Graphene/Ru(0001); and (top spectrum) oxidized Graphene/Ru(0001) after annealing to 1000 K. The graphene layer was produced by four ethylene exposure cycles as described in the text.

XPS spectra recorded after annealing to 1000 K are shown in Figure 7. It is observed that the C1s emission still remains after annealing, in agreement with the LEED results in Figure 1d which also shows that graphene is still present after annealing. Figure 7 shows C1s and Ru3d XPS after formation of graphene and after annealing to 1000 K of the oxidized samples, respectively. In Figure 7a,b are shown results in the case of 2 ethylene exposure cycles, and Figure 7c,d show similar spectra for 4 ethylene exposure cycles. The estimated thickness of the graphene layer is reduced by about 38% and 48% after annealing to 1000 K of the oxidized samples in the case of 2 and 4 ethylene exposure cycles, respectively. Figure 8 shows the absence of O1s emission in the XPS after annealing to 1000 K in agreement with previous results [14], where it was found that oxidation of the graphene on Ru system only takes place at elevated temperatures above 600 K. The graphene layer is stable at temperatures at least up to 1250 K (as investigated in this study) in the absence of oxygen atoms at the interface. For graphene on a Ru system that had been oxidized at 675 K, the situation is different; the carbon atoms from the graphene layer react with oxygen atoms at the Ru surface and desorb as CO and CO${}_{2}$. When all the oxygen atoms have reacted with carbon atoms and desorbed, an incomplete graphene layer is left on the Ru surface.

In the case of one ethylene exposure cycle, that is, an incomplete graphene layer, a very sharp CO TPD peak is observed at about 780 K (top spectrum of Figure 5a). The small features on the right hand side of the sharp desorption peak are believed to be instrumental, and are assumed to be caused by an inability of the spectrometer to follow the rapid rate of change of the pressure increase/decrease. It is noted that the general shape of the desorption spectrum from the incomplete graphene layer was reproduced several times, which verifies the abrupt nature of the desorption. For 4 and 6 ethylene exposure cycles wider TPD spectra having peaks near 730 K and near 800 K were observed (middle and bottom spectra in Figure 5a). With the onset of CO${}_{2}$ desorption for an incomplete graphene layer, the top spectrum of Figure 5b shows a less steep initial desorption characteristics which subsequently changes to a very steep desorption curve as the maximum of the desorption spectrum is approached. It is observed for the incomplete graphene layer that a desorption of CO${}_{2}$ is initiated before the desorption of CO. This may indicate that the reaction between two oxygen atoms and one carbon atom starts the decomposition of the incomplete graphene layer. Eventually for increasing temperature, reaction of carbon and oxygen and subsequent desorption of carbon monoxide dominates.

### 2.3. TPD Analysis

A main goal of TPD studies is to determine the kinetic parameters for desorption from crystalline surfaces, that is, the order of desorption, the desorption energy, and the desorption prefactor. The desorption rate *r* for activated desorption from a surface may, according to Redhead [46], be described by the Polanyi–Wigner formulation of reaction kinetics:

(1) $$r\left(t\right)=-dn/dt=\nu n^{k}e^{-E_{d}/k_{B}T}$$

where *n* is the surface coverage (number of atoms per unit volume), ν is the desorption prefactor, *k* is the order of the desorption, $E_{d}$ is the desorption energy, and *T* is the temperature. In general the desorption process is made complicated by desorption from lattice sites of different adsorption energies, and by that both the prefactor and desorption energy in general depend on the surface coverage as well as lateral interactions between adsorbates [47]. First-order desorption describes desorption of adsorbed molecules, and second-order desorption describes recombinative desorption, for example, C and O atoms recombine and desorb as CO. Even noninteger orders of desorption may occur due to strong lateral interactions, multiple adsorption sites, and nonrandom adsorption sites. The present case where carbon and oxygen atoms recombine to desorb as CO and to a lesser extent as CO${}_{2}$ would be a candidate for second-order desorption.

Methods of extracting desorption parameters may be grouped in (1) the integral approach where peak characteristics like temperature at peak maximum and half-width are used, and (2) the differential approach where desorption rate and temperature pairs obtained from several TPD spectra are used in an Arrhenius plot where the slope and intercept give the desorption parameters. The latter method must be used for coverage-dependent desorption parameters. It has been demonstrated that for high coverage the threshold TPD method may give reliable values for desorption parameters in a convenient way [48]. The coverage in a TPD spectrum is proportional with the area under the desorption curve, and by analyzing the low temperature onset of the spectrum in a narrow temperature region the coverage may be assumed to be approximately constant. The desorption energy may be extracted without knowing the order of the desorption (i.e., molecular or recombinative desorption). This method is useful when the kinetic parameters vary with coverage. In particular, this method may be applied confidently in cases of several overlapping desorption peaks [48].

A simplified analysis of the TPD data may be performed by considering the temperature dependence of the desorption rate r(T) in the following form:

(2) $$r\left(T\right)=A\left(n\right)e^{-E_{d}/k_{B}T}$$

where the prefactor A generally depends on the surface coverage *n*. The desorption rate may be thus written as:

(3) $$ln\left(r\right)=-\frac{E_{d}}{k_{B}T}+ln\left(A\right)$$

**Figure 9:**
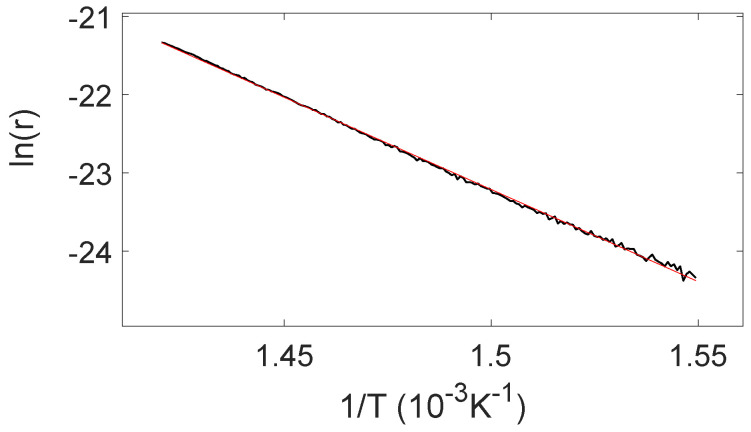
The logarithm of the desorption rate plotted versus 1/T for desorption of CO from a sample of six ethylene exposure cycles. The straight line is a linear fit to the data over a narrow temperature region and is used to estimate desorption parameters.

In a narrow temperature region of the low temperature onset of the desorption spectrum where the prefactor A(n) is approximately constant (i.e., small change in coverage) the desorption energy may be estimated by the linear slope of the curve as ln(r) is plotted as a function of 1/T. This is shown in Figure 9 in the case of six ethylene exposure cycles. The fitted temperature range was from 650 to 700 K and the desorption energy was estimated to $E_{d}\approx$ 2.0 eV. To obtain an estimate for the desorption prefactor the mass spectrometer signal must be normalized to the surface coverage. For surface coverage measured as a fraction of a monolayer θ the desorption rate may be written:

(4) $$r\left(t\right)=-d\theta /dt=\nu \theta^{k}e^{-E_{d}/k_{B}T}$$

If the initial coverage is one monolayer θ=1, and if the coverage is nearly constant in the fitted region the desorption rate may be written:

(5) $$ln\left(r/\int_{T_{min}}^{T_{max}}rdT\right)=-\frac{E_{d}}{k_{B}T}+ln\left(\nu \right)$$

By using this procedure, a prefactor of $\nu \approx 2\cdot 10^{5}$ s${}^{-1}$ is obtained in the case of six exposure and annealing cycles. In this case, a complete graphene layer had been formed on the Ru(0001) crystal. This procedure does not determine the order of the desorption process. The value of the prefactor is uncertain since the concept of coverage may not be clearly defined in this system where oxygen atoms at the ruthenium interface react with carbon atoms in the graphene layer above to desorb as CO molecules. The lower than usual value of the prefactor as compared to the typical value of ≈10${}^{13}$ s${}^{-1}$ for first-order desorption, may presumably be caused by the relative complicated nature of the desorption process.

In the case of one exposure cycle, that is, an incomplete graphene layer, a linear region was not found when ln(r) was plotted versus 1/T. It is concluded that the very steep desorption curve which was obtained in the case of 1 exposure cycle, top curve of Figure 5a, may not be analyzed by using this method. This may be caused by the fact that the rapid desorption violates the criterion of a nearly constant coverage over a narrow temperature region. For the incomplete graphene layer the desorption of CO from the oxidized sample shows a different characteristic with a very sharp desorption peak. Rapid reaction of oxygen and carbon at the Ru surface seemingly results in an abrupt desorption of CO at about 50 K higher temperature as compared to the case of a complete graphene layer. This behaviour was observed repeatedly in this carbon coverage regime. For the incomplete graphene layer the Ru-C interaction should be of more importance for the desorption characteristics as compared to the samples of complete graphene layers since lateral interactions between carbon atoms would be reduced.

## 3. Materials and Methods

All measurements were performed in situ in an ultra-high-vacuum chamber of base pressure about $1\cdot 10^{-8}$ Pa. The chamber is equipped with an ion sputtering gun (PSP Instruments) for sample cleaning, a LEED optics (SPECS), a mass-spectrometer for TPD measurements, as well as facilities for photoelectron spectroscopy. The sample could be measured by all these techniques without sample transfer. The measurement sample could be cooled by liquid nitrogen to 100 K, and could be heated to temperatures above 1300 K. XPS and UPS measurements were recorded using a SES2002 spectrometer (Scienta) in conjunction with a monochromatized Al Kα X-ray source of photon energy hν = 1486.6 eV (Scienta) and a UVS300 He gas discharge lamp (Specs) which provided photons of energy hν = 21.2 eV. The photoelectrons where collected in a cone of angle about 30°. The experimental energy resolution of the XPS was about 0.4 eV at an electron pass energy of 200 eV. For the UPS measurements an energy resolution of 20 meV was obtained at pass energy 15 eV. Since the analyser and sample Fermi levels are in electrical contact with each other the sample work function could be estimated from the position of the low kinetic energy cut-off of the UPS spectra when using a sample bias of −5 V. The polished Ru(0001) crystal was obtained from MaTeck Material Technologie & Kristalle GmbH, and was cleaned by repeated Ar sputtering at 900 K, flash heating to 1250 K, and oxygen annealing at 900 K. The quality of the Ru crystal was verified by XPS and LEED. Graphene was deposited on the Ru substrate by dosing ethylene (C_2_H_4_) at partial pressure $1.33\cdot 10^{-5}$ Pa for 100 s at room temperature followed by annealing to 1250 K. This procedure was repeated several times to obtain a saturated graphene layer. The ethylene gas was of purity 3N (99.9%) and was supplied by Linde Gas. Argon and oxygen gases were of purity 6N and 5N, respectively. TPD spectra were obtained by using a shielded and differentially pumped Prisma Plus quadrupole mass spectrometer (Pfeiffer). In the limit of large pumping speed the desorption rate is proportional to the change in partial pressure of the desorbing molecule. The present vacuum system satisfies this requirement; the vacuum systems is pumped by two 450 L/s Leybold turbo pumps in addition to a 600 L/s ion pump from Physical Electronics. The mass spectrometer was differentially pumped by a 600 L/s Leybold turbo pump. The mass spectrometer was fitted on a linear motion drive and was positioned close to the sample surface during measurements to discriminate against spurious desorption from the sample support, edges and back side of the sample, and to obtain reproducible intensities that could be compared for different runs. Spectra were obtained from masses 2, 18, 28, 32, and 44 u simultaneously for all TPD runs. The mass-spectrometer was thoroughly degassed before experiments to reduce contributions from background gases in the mass spectrometer confinement. The temperature rate was kept at 1 K/s during the TPD measurements.

## 4. Conclusions

The stability of intercalated oxygen at the interface between graphene and Ru(0001) was investigated by TPD, XPS, UPS, and LEED. It was observed that the intercalated oxygen atoms interacted with the graphene layer and desorbed as CO and to a lesser extent as CO${}_{2}$ at temperatures in the range 700 to 850 K. No O${}_{2}$ desorption was observed contrary to previous claims in the literature. It was found that patches of the graphene layer were intact after the desorption run and that the intercalated oxygen was removed. The graphene layer was found to disintegrate at temperatures above 700 K as long as oxygen atoms were available to react to CO and CO${}_{2}$.

## Data Availability

The data presented in this study are available on request from the corresponding author.
